# Supplementary material for: Live software documentation of design pattern instances
Source: PeerJ Comput Sci. 2024 Aug 16;10:e2090. doi: 10.7717/peerj-cs.2090 (PMC11419627; doi:10.7717/peerj-cs.2090)
Supplement: Supplemental Information 1 [file peerj-cs-10-2090-s001.pdf]

Design Pattern Doc Questionnaire

* Required

Background Check

This work is centered around the Design patterns from the "Gang of Four", being them Factory Method, Builder, Strategy, Observer, Command, and so on.

1. At this point ... *

Mark only one oval per row.

	Strongly Disagree	Disagree	Neutral	Agree	Strongly Agree
... I'm comfortable working with Intellij	◯	◯	◯	◯	◯
... I'm comfortable programming in Java	◯	◯	◯	◯	◯
... I know well the GoF design patterns	◯	◯	◯	◯	◯
... I can recognize GoF design patterns in code	◯	◯	◯	◯	◯
... I can implement GoF design patterns	◯	◯	◯	◯	◯
... I'm comfortable working with draw.io	◯	◯	◯	◯	◯

2. Gender *

Mark only one oval.

◯ Female
◯ Male
◯ Prefer not to say

3. What is the highest degree or level of education you have completed? *

Mark only one oval.

◯ High School
◯ Bachelor's degree
◯ Master's degree
◯ Doctoral degree

4. If you are a student from MIEIC, what is your current year?

Mark only one oval.

- ( ) 1st
- ( ) 2nd
- ( ) 3rd
- ( ) 4th
- ( ) 5th

Tasks

During this part of the experiment, you will be submitted to a couple of tasks. For each task, you will be given a different source code folder. Note that, at the end of the third task, you will be asked to submit your solutions (more details on how to do this will be discussed later). The experiment will take approximately 40-50 minutes. It is important to do it in a single run, without interruptions.

Some tasks involve submitting class diagrams showing the implementation of pattern instances. An example for this type of task can be seen in the figure below. Note that, we are using stereotypes (<<Role>>), which are not the standard format, for representing the pattern roles. For this type of tasks, we would like you to use: https://draw.io

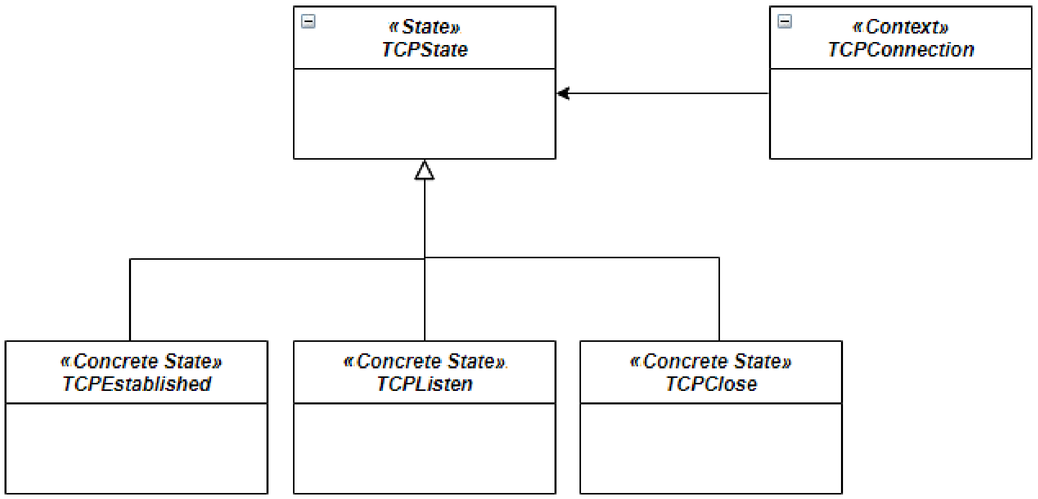

We suggest to take a quick look at the State design pattern, on the cheat sheet provided, in order to easily comprehend how both diagrams relate to each other. As part of the experiment, you will be given access to the development environment Intellij.

Task 1

5. 🕑 Before starting this task, please write down the current time: *

Example: 8:30 AM

6. T11 This source code contains one or more pattern instances. Which design pattern(s) are represented in the system? *

7. T12 Document the pattern instances that you have found as a UML class diagram. Do it as was instructed previously, using draw.io, and specifying pattern roles as class stereotypes

   Files submitted:

8. 🕑 After you have finished this task, please write down the current time: *

Example: 8:30 AM

Task 2

9. 🕑 Before starting this task, please write down the current time: *

Example: 8:30 AM

10. T21 John is trying to implement a simple system for controlling the light of a lightbulb, in his house. Unfortunatelly, he can't get it to work. Which pattern participant(s) are missing? *

T22 Create new objects and/or modify those already provided to complete the system.

11. 🕑 After you have finished this task, please write down the current time: *

Example: 8:30 AM

Task 3

The zookeepers from Maia's Zoo have designed a system to easily manage their animal's diet and daily feeding times. Upon the arrival of a new specie to the zoo, the system must be updated accordingly. Today, a specie of Giraffe has arrived to the zoo. Can you help the zookeepers introducing it to the system?

12. 🕑 Before starting this task, please write down the current time: *

Example: 8:30 AM

13. T31 Identify the main GoF pattern in this code and explain what changes (objects, pattern roles) would you need to apply to the system to contemplate these requirements *

14. T32 Implement those changes and add the pattern instances documentation required to understand the extended system. (Here submit only the class diagrams)

Files submitted:

15. 🕑 After you have finished this task, please write down the current time: *

Example: 8:30 AM

16. Create a folder containing the folders: 1) task2 and 2) task3. Inside those folders place the respective source code that resulted from your solution to the tasks. Zip the folder and submit it below:

Files submitted:

Final feedback

17. Select your opinion towards the following sentences: *

Mark only one oval per row.

	Strongly Disagree	Disagree	Neutral	Agree	Strongly Agree
I found it easy to identify design patterns in the source code	⬭	⬭	⬭	⬭	⬭
I found it easy to document the code using pattern instances in UML format	⬭	⬭	⬭	⬭	⬭
The communication environment (remote computer) had a negative impact in the experiment	⬭	⬭	⬭	⬭	⬭

This content is neither created nor endorsed by Google.

Google Forms
