# Supplementary material for: Live software documentation of design pattern instances
Source: PeerJ Comput Sci. 2024 Aug 16;10:e2090. doi: 10.7717/peerj-cs.2090 (PMC11419627; doi:10.7717/peerj-cs.2090)
Supplement: Supplemental Information 2 [file peerj-cs-10-2090-s002.pdf]

Design Pattern Doc Questionnaire

* Required

Background Check

This work is centered around the Design patterns from the "Gang of Four", being them Factory Method, Builder, Strategy, Observer, Command, and so on.

1. At this point ... *

Mark only one oval per row.

	Strongly Disagree	Disagree	Neutral	Agree	Strongly Agree
... I'm comfortable working with Intellij	⬭	⬭	⬭	⬭	⬭
... I'm comfortable programming in Java	⬭	⬭	⬭	⬭	⬭
... I know well the GoF design patterns	⬭	⬭	⬭	⬭	⬭
... I can recognize GoF design patterns in code	⬭	⬭	⬭	⬭	⬭
... I can implement GoF design patterns	⬭	⬭	⬭	⬭	⬭

2. Gender *

Mark only one oval.

- ⬭ Female
- ⬭ Male
- ⬭ Prefer not to say

3. What is the highest degree or level of education you have completed? *

Mark only one oval.

- ⬭ High School
- ⬭ Bachelor's degree
- ⬭ Master's degree
- ⬭ Doctoral degree

4. If you are a student from MIEIC, what is your current year?

Mark only only one oval.

- 1st
- 2nd
- 3rd
- 4th
- 5th

Tasks

During this part of the experiment, you will be submitted to a couple of tasks. For each task, you will be given a different source code folder. Note that, at the end of the third task, you will be asked to submit your solutions (more details on how to do this will be discussed later). The experiment will take approximately 40-50 minutes. It is important to do it in a single run, without interruptions.

Some tasks involve submitting class diagrams showing the implementation of pattern instances. An example for this type of task can be seen in the figure below. Note that, we are using stereotypes (<<Role>>), which are not the standard format, for representing the pattern roles. For this type of tasks, we would like you to use the plugin discussed on the following section.

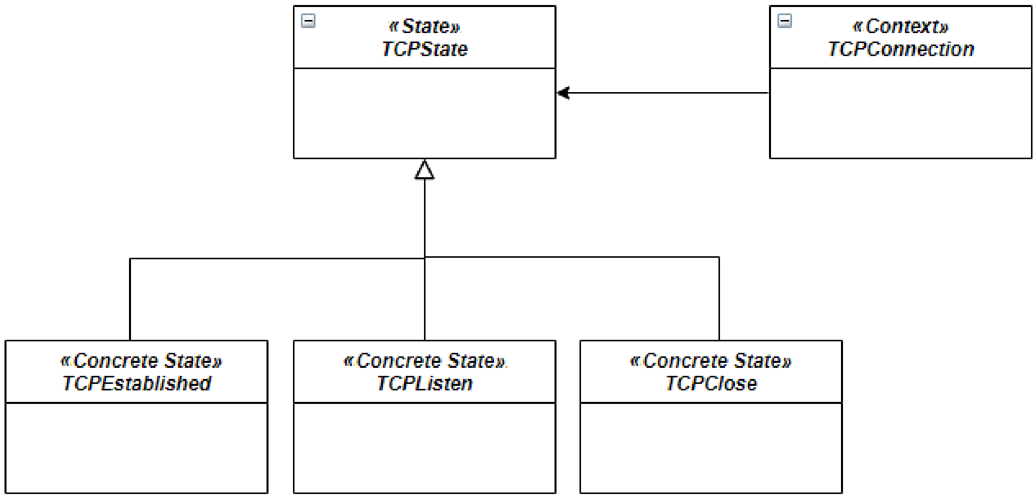

We suggest to take a quick look at the State design pattern, on the cheat sheet provided, in order to easily comprehend how both diagrams relate to each other.

Plugin Walkthrough

As part of the experiment, you will be given access to the development environment Intellij with a plugin for documenting pattern instances. For that reason, we would like to highlight its main features and explain how to take advantage of them. We will start by introducing those that allow creating/updating the documentation:

Feature 1 - Pattern instance documentation suggestion. This plugin uses a design pattern detection tool to scan the project for design patterns. Those detected by it are highlighted as can be seen in the figure below (background yellow-brownish color). Accepting the suggestion will persist the pattern instance documentation for that design pattern.

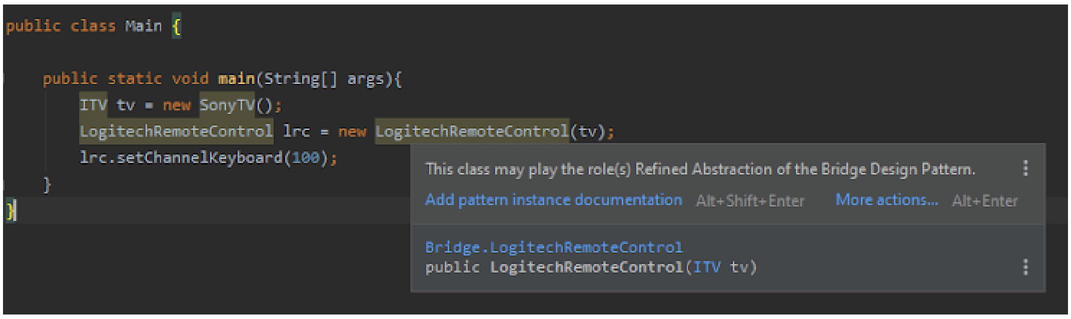

Feature 2 - Manually document pattern instance. By right clicking on a object, a default pattern instance is created for that object.

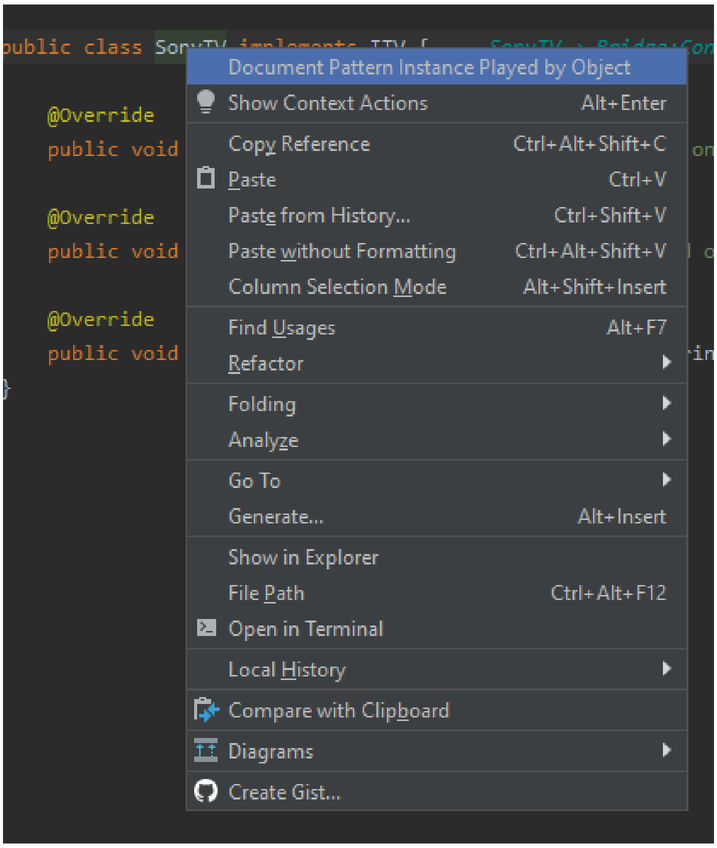

Feature 3 - Manually edit documentation. When the cursor is on top of an object, its documentation is displayed on the pattern instance documentation editor (window on the right side of intellij editor). This is a live editor of the pattern instances played by that object.

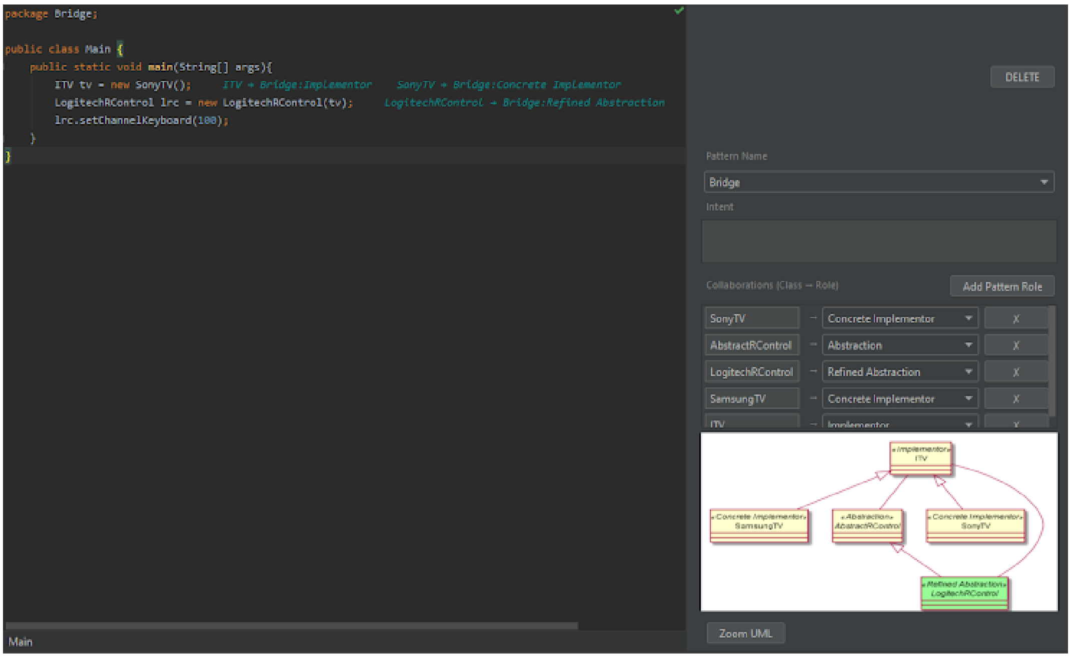

Feature 4 - Incomplete implementation warning. When the code is not consistent with its pattern instance documentation (eg. pattern instance role is missing), objects that play a part in that pattern instance are highlighted. Users can decide whether they would like to edit the documentation or delete it.

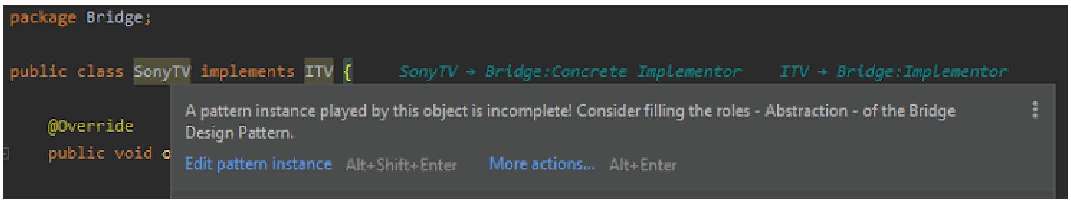

The following features are merely visual. Their purpose is to display the persisted pattern instance documentation:

Feature 5 - Pattern Hints (text at the end of the line where pattern participants are found). These provide information regarding played roles in persisted pattern instances.

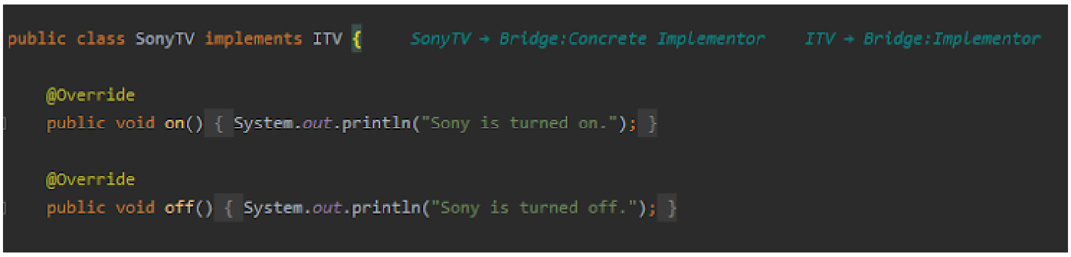

Feature 6 - Pattern documentation. When hovering a pattern participant, a pop up dialog appears with the UML pattern instances documentation, played by that object.

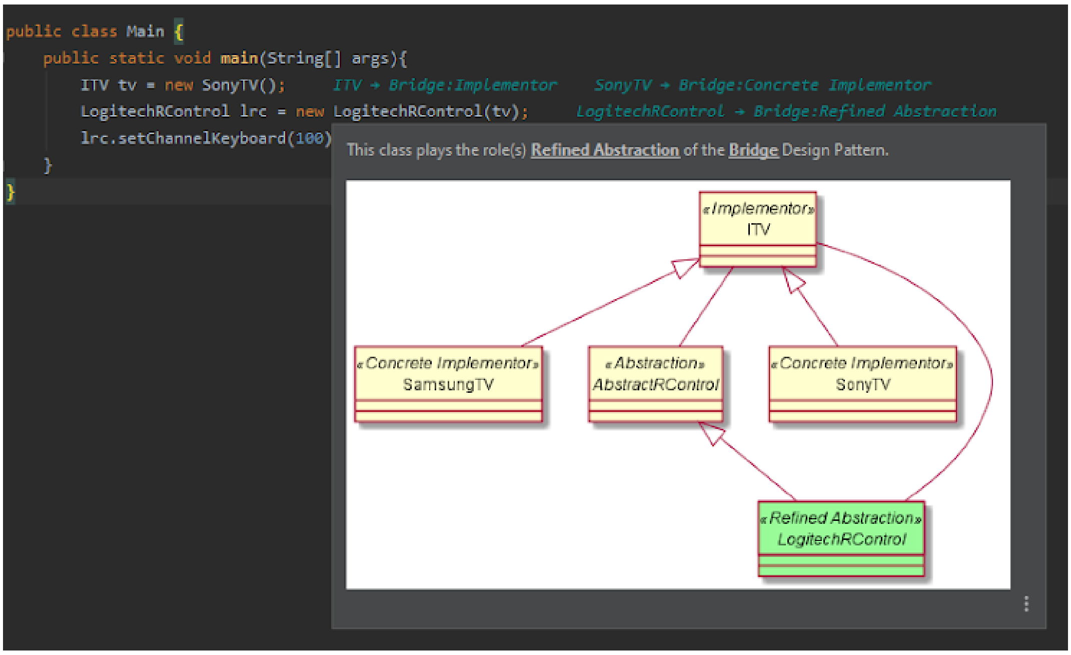

Task 1

5. 🕑 Before starting this task, please write down the current time: *

Example: 8:30 AM

6. T11 This source code contains one or more pattern instances. Which design pattern(s) are represented in the system? *

7. T12 Document the pattern instances that you have found as a UML class diagram. Do it as was instructed previously, using the plugin, and specifying pattern roles as class stereotypes.

Files submitted:

8. 🕑 After you have finished this task, please write down the current time: *

Example: 8:30 AM

Task 2

9. 🕑 Before starting this task, please write down the current time: *

Example: 8:30 AM

10. T21 John is trying to implement a simple system for controlling the light of a lightbulb, in his house. Unfortunatelly, he can't get it to work. Which pattern participant(s) are missing? *

T22 **Create new objects and/or modify those already provided to complete the system.**

11. 🕑 After you have finished this task, please write down the current time: *

Example: 8:30 AM

Task 3

The zookeepers from Maia's Zoo have designed a system to easily manage their animal's diet and daily feeding times. Upon the arrival of a new specie to the zoo, the system must be updated accordingly. Today, a specie of Giraffe has arrived to the zoo. Can you help the zookeepers introducing it to the system?

12. 🕑 Before starting this task, please write down the current time: *

Example: 8:30 AM

13. T31 Identify the main GoF pattern in this code and explain what changes (objects, pattern roles) would you need to apply to the system to contemplate these requirements *

14. T32 Implement those changes and add the pattern instances documentation required to understand the extended system. (Here submit only the documentation)

Files submitted:

15. 🕑 After you have finished this task, please write down the current time: *

____

Example: 8:30 AM

16. Create a folder containing the folders: 1) task2 and 2) task3. Inside those folders place the respective source code that resulted from your solution to the tasks. Zip the folder and submit it below:

Files submitted:

Final feedback

17. Select your opinion towards the following sentences: *

Mark only one oval per row.

	Strongly Disagree	Disagree	Neutral	Agree	Strongly Agree
I found it easy to identify design patterns in the source code	⬭	⬭	⬭	⬭	⬭
I found it easy to document the code using pattern instances in UML format	⬭	⬭	⬭	⬭	⬭
The communication environment (remote computer) had a negative impact in the experiment	⬭	⬭	⬭	⬭	⬭

18. The pattern hints (text in front of each pattern participant line) helped ... *

```
public class Horse extends Animal{   Horse → Abstract Factory:Product  Animal → Abstract Factory:Abstract Product
```

Mark only one oval per row.

	Strongly Disagree	Disagree	Neutral	Agree	Strongly Agree
... understanding the code	⬭	⬭	⬭	⬭	⬭
... solving the tasks quicker	⬭	⬭	⬭	⬭	⬭
... registering and keeping the pattern instance information updated	⬭	⬭	⬭	⬭	⬭

19. The documentation provided when hovering on top of the pattern participants helped ... *

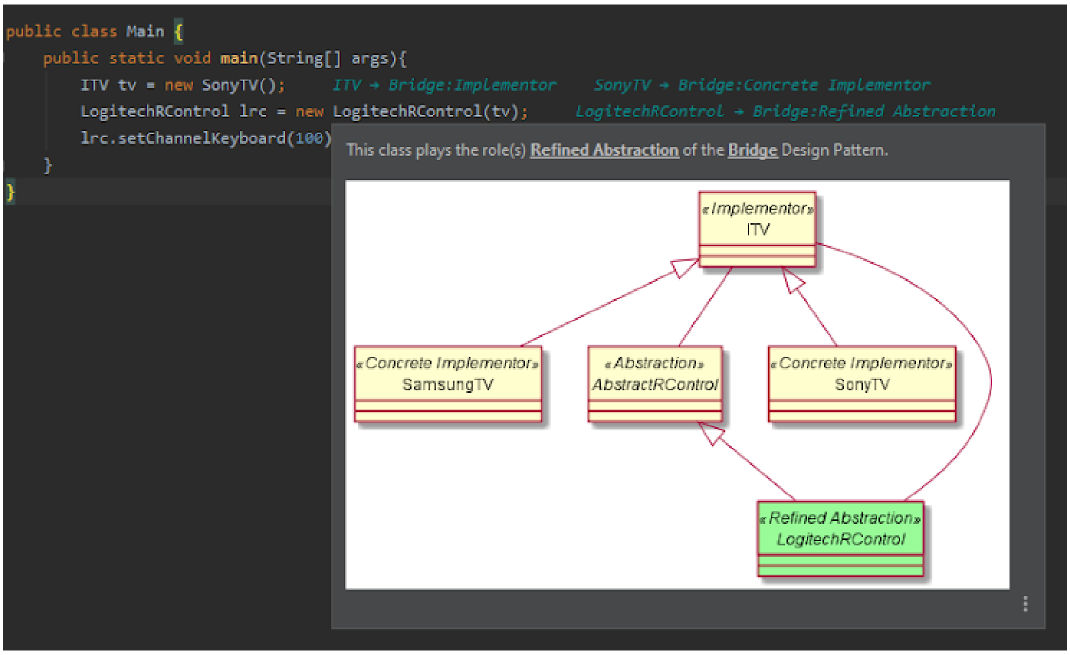

Mark only one oval per row.

	Strongly Disagree	Disagree	Neutral	Agree	Strongly Agree
... understanding the code					
... solving the tasks quicker					
... registering and keeping the pattern instance information updated					

20. The highlighting of pattern participants when suggesting design patterns helped ... *

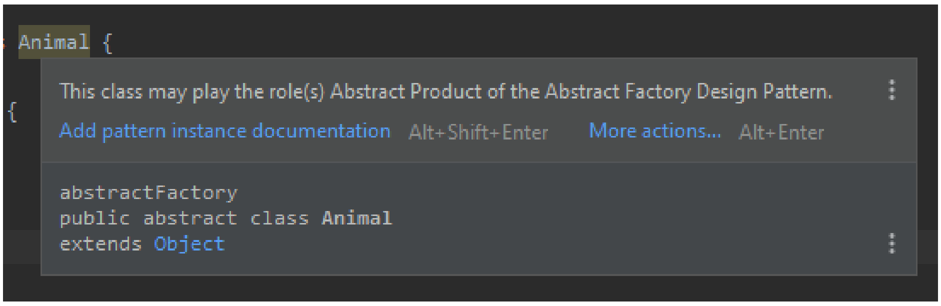

Mark only one oval per row.

	Strongly Disagree	Disagree	Neutral	Agree	Strongly Agree
... understanding the code					
... solving the tasks quicker					
... registering and keeping the pattern instance information updated					

21. The highlighting of pattern participants, when some roles are missing regarding the documentation, helped ... *

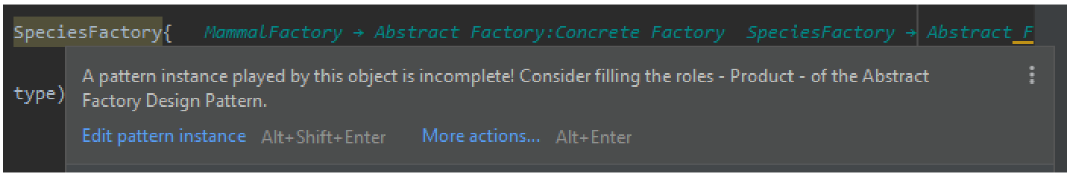

Mark only one oval per row.

	Strongly Disagree	Disagree	Neutral	Agree	Strongly Agree
... understanding the code					
... solving the tasks quicker					
... registering and keeping the pattern instance information updated					

22. The live update of documentation helped ... *

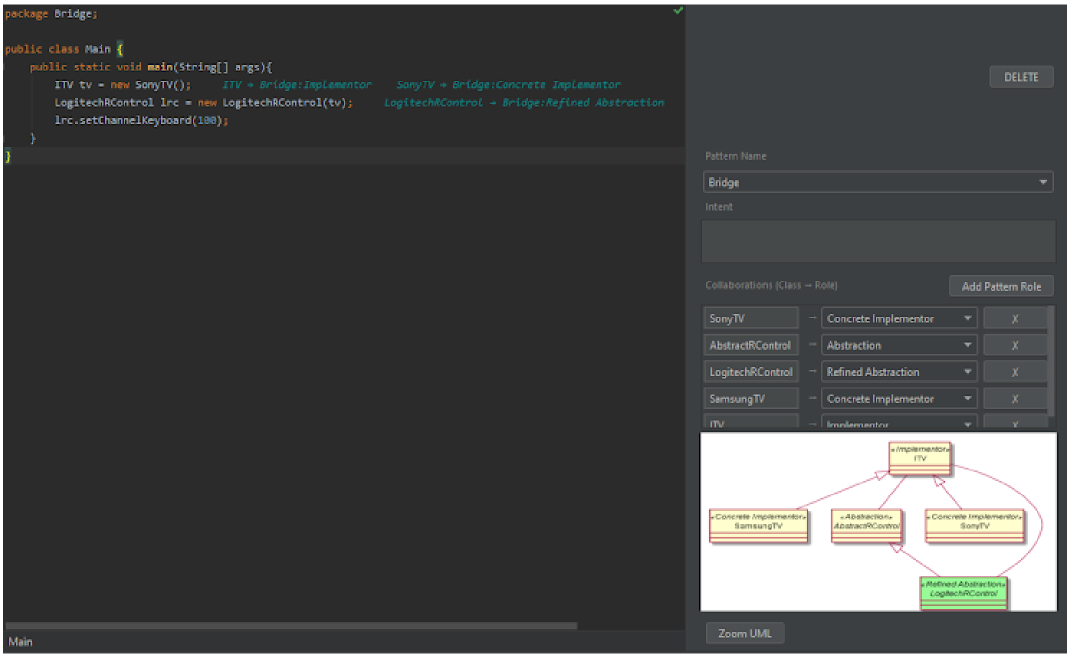

Mark only one oval per row.

	Strongly Disagree	Disagree	Neutral	Agree	Strongly Agree
... understanding the code					
... solving the tasks quicker					
... registering and keeping the pattern instance information updated					

This content is neither created nor endorsed by Google.

Google Forms
